# Thermo-Viscoelastic Response of Protein-Based Hydrogels

**DOI:** 10.3390/bioengineering8060073

**Published:** 2021-05-31

**Authors:** Aleksey D. Drozdov, Jesper deClaville Christiansen

**Affiliations:** Department of Materials and Production, Aalborg University, Fibigerstraede 16, 9220 Aalborg, Denmark; jc@mp.aau.dk

**Keywords:** protein-based gels, recombinant proteins, coiled coil complexes, viscoelasticity

## Abstract

Because of the bioactivity and biocompatibility of protein-based gels and the reversible nature of bonds between associating coiled coils, these materials demonstrate a wide spectrum of potential applications in targeted drug delivery, tissue engineering, and regenerative medicine. The kinetics of rearrangement (association and dissociation) of the physical bonds between chains has been traditionally studied in shear relaxation tests and small-amplitude oscillatory tests. A characteristic feature of recombinant protein gels is that chains in the polymer network are connected by temporary bonds between the coiled coil complexes and permanent cross-links between functional groups of amino acids. A simple model is developed for the linear viscoelastic behavior of protein-based gels. Its advantage is that, on the one hand, the model only involves five material parameters with transparent physical meaning and, on the other, it correctly reproduces experimental data in shear relaxation and oscillatory tests. The model is applied to study the effects of temperature, the concentration of proteins, and their structure on the viscoelastic response of hydrogels.

## 1. Introduction

The design, preparation, and analysis of the mechanical response and physical and biological properties of hydrogels based on recombinant proteins and synthetic peptides have attracted considerable attention in the past decade [1,2,3,4,5,6,7,8]. Because of the reversible nature of bonds that formed by associating coiled-coil domains [9,10], these gels demonstrate important features, such as (i) self-assembling [11], (ii) thixotropic behavior and injectability [12], (iii) self-healing ability [13], (iv) shape memory [14], and (v) strong adhesion to biological tissues [15].

An advantage of engineered protein gels as compared with synthetic polymer gels is that they: (i) are biocompatible, biodegradable, non-immunogenic, non-toxic, and responsive to various biological stimuli [16], (ii) recombinant proteins and synthetic peptides have highly homogeneous lengths, sequences, and compositions of blocks (that allows inhomogeneities to be avoided that arise in conventional gels due to the non-uniform distribution of chains with various molecular weights [4]), and (iii) owing to the modular nature of artificial proteins and peptides, they can be used as building blocks for designing stimuli-responsive hydrogels with required micro-structures and properties [17]. An advantage of recombinant proteins when compared with those extracted from natural sources is that (i) concerns that are caused by disease transmission, immunogenic responses, and large batch-to-batch variability do not arise for these materials [18], while (ii) their physical properties and biological activity can be modulated by changing the biosynthesis conditions [19].

Because of the bioactivity of protein- and peptide-based gels and their superior mechanical and physical properties (that resemble those of the extracellular matrix), these materials have shown promise for applications in targeted drug delivery [20] and localized viral gene delivery [21], bioimaging [22], biosensing [23], bioelectronics [24], vaccine engineering [25], wound healing [26], therapy [27], as well as tissue engineering and regenerative medicine [16,28].

The development of new routes for manufacturing artificial protein and peptide gels and the design of hydrogels with improved functional properties require a deep understanding of correlations between the mechanical behavior of these materials at the macro-scale, and sequences of primary amino acids, covalent and non-covalent interactions between individual amino acids and secondary structures formed by their blocks at the micro-scale [29,30,31]. Linear and nonlinear rheology (shear oscillatory tests with small and large amplitudes, shear relaxation and creep tests, etc.) provides a convenient tool for studying the effects of external factors (temperature, pH, and ionic strength of solutions) on the concentration of chemical and physical cross-links, inhomogeneity of their distribution in a gel, and the characteristic rates for the dissociation and re-association of temporary bonds [32,33]. Rheological analysis of the viscoelastic properties of hydrogels with supramolecular and dynamic covalent bonds has recently attracted considerable attention, as it allows (i) the shear-thinning and injectability of these gels to be evaluated [34], and (ii) cell attachment, migration, proliferation, and differentiation in hydrogel scaffolds to be controlled [35,36].

The rearrangement (dissociation and re-association) of temporary bonds between chains drives the viscoelastic response of supramolecular gels. The kinetics of the rearrangement process is conventionally studied by means of the small amplitude shear oscillatory tests in the frequency sweep mode [37]. Because of the variety of supramolecular motifs (hydrogen bonding, hydrophobic interaction, multivalent electrostatic interaction, dipole-dipole interaction, metal-ligand coordination, host-guest recognition, etc. [38]), a direct comparison of observations in these tests is difficult, and more sophisticated treatment of the experimental data is required [39].

The effects of the angular frequency of oscillations ω on the storage $G^{\prime }$ and loss $G^{\prime \prime }$ moduli of a supramolecular gel are traditionally described by means of the generalized Maxwell model with three material constants: a shear modulus μ, an average characteristic time for stress relaxation $\tau_{0}$, and a dimensionless measure of distribution of relaxation times Σ. According to this model,

(1) $$G^{\prime }\left(\omega \right)=\mu \int_{0}^{\infty }H\left(\tau \right)\frac{\omega^{2}\tau^{2}}{1+\omega^{2}\tau^{2}}d\ln\tau ,\;G^{\prime \prime }\left(\omega \right)=\mu \int_{0}^{\infty }H\left(\tau \right)\frac{\omega \tau }{1+\omega^{2}\tau^{2}}d\ln\tau ,$$

where H(τ) stands for the relaxation spectrum. The log-normal expression is adopted for the function H(τ),

(2) $$H\left(\tau \right)=H_{0}\exp\left[-\frac{{\left(\ln\tau -\ln\tau_{0}\right)}^{2}}{2\Sigma^{2}}\right],$$

where the pre-factor $H_{0}$ is determined from the normalization condition

$$\int_{0}^{\infty }H\left(\tau \right)d\ln\tau =1.$$

An advantage of Equations (1) and (2) is that they only involve three adjustable parameters (which implies that these relations can be applied for a comparison of the viscoelastic responses of hydogrels with various chemical structures and compositions). Their shortcoming is that Equations (1) and (2) cannot reproduce an upturn of the graph $G^{\prime \prime }\left(\omega \right)$ that was observed at relatively large frequencies. In order to improve the accuracy of fitting observations, several modifications of these equations were suggested in [40,41,42,43,44].

Although artificial protein- and peptide-based gels belong to a larger family of supramolecular hydrogels [45], two characteristic features distinguish their viscoelastic response:

1. Polymer chains in protein and peptide gels are connected by (i) temporary bonds between the secondary structures of their blocks (specific protein–protein and protein–peptide interactions [46]), and (ii) covalent cross-links between functional groups of amino acids [47] (which implies that Equation (1) is to be modified to account for the presence of permanent bonds between chains).
2. When the physical cross-links between protein and peptide motifs are sufficiently strong and their rates of dissociation are low under physiological conditions (which implies that the moduli $G^{\prime }$ and $G^{\prime \prime }$ become practically independent of ω in the conventional interval of frequencies between 0.1 and 100 rad/s [48]), the kinetics of dissociation of temporary bonds is evaluated by combining the experimental data in shear oscillatory tests, on the one hand, and shear relaxation [49] or creep [50] tests, on the other.

A model is required that describes observations in a unified manner in order to analyze the experimental data in these tests. However, conventional models treat these data separately: observations in oscillatory tests are predicted by Equations (1) and (2), whereas the decay in the relaxation modulus is described by the Kohlrausch stretched exponential function.

The objective of this study is threefold: (i) to develop a simple model (with a small number of material constants) in the linear thermo-viscoelasticity of protein-based gels, (ii) to demonstrate its ability to describe the experimental data in small-amplitude shear oscillatory tests, relaxation tests, and creep tests in a unified manner, and (iii) to find material parameters by fitting observations on gels with various architectures, chemical compositions, and concentrations of proteins in the entire interval of physiological temperatures.

The exposition is organized, as follows. Section 2 develops a model for the linear viscoelastic response of hydrogels whose chains are bridged by permanent and temporary cross-links. Observations in small-amplitude shear oscillatory tests and relaxation tests on biocompatible gels are analyzed in Section 4 and are extended to protein-based gels in Section 5. Concluding remarks are formulated in Section 6 on biocompatible gels and protein-based gels are analyzed in Section 4 and Section 5.

## 2. Model

A gel is modeled as a two-phase medium that is composed of an equivalent polymer network and water molecules. The deformation of the network coincides with the macro-deformation of the gel (the affine hypothesis).

The network consists of two types of chains: permanent (both of their ends are connected to separate junctions formed by covalent cross-links) and temporary (at least one end of these chains is connected to a physical bond that can dissociate and re-associate) [51,52]. Two states of a temporary chain are distinguished: (i) active (both ends of the chain are connected to the network) and (ii) dangling (an end of a chain detaches from the network due to the dissociation of an appropriate bond). The chain is transformed into the dangling state when an end of an active chain separates from the network at some instant $\tau_{1}$. When the free end of the dangling chain merges with the network at an instant $\tau_{2}>\tau_{1}$, the chain returns into the active state. Attachment and detachment events occur at random times being driven by thermal fluctuations.

The network is presumed to be inhomogeneous: it consists of meso-domains with various activation energies *u* for rearrangement of bonds. The Eyring equation gives the rate of the breakage of bonds (the separation of active chains from their junctions) in a meso-domain with activation energy *u*

$$\Gamma =\Gamma_{0}\exp\left(-\frac{u}{k_{B}T_{0}}\right),$$

where $\Gamma_{0}$ is the attempt rate, $T_{0}$ stands for a fixed temperature, and $k_{B}$ is the Boltzmann constant. It follows from this relation that

(3) $$\Gamma \left(v\right)=\Gamma_{0}\exp\left(-v\right),$$

where $v=u/(k_{B}T_{0})$ stands for the dimensionless activation energy.

The probability density f(v) to find a meso-domain with activation energy v≥0 characterizes the inhomogeneity of the network. With reference to the random energy model [53], the quasi-Gaussian formula is adopted,

(4) $$f\left(v\right)=f_{0}\exp\left(-\frac{v^{2}}{2\Sigma^{2}}\right).$$

An advantage of Equation (4) is that the distribution of meso-domains is determined by the only parameter Σ>0, while the pre-factor $f_{0}$ is found from the normalization condition

(5) $$\int_{0}^{\infty }f\left(v\right)dv=1.$$

At an arbitrary instant t≥0, the ensemble of temporary chains is entirely characterized by the function n(t,τ,v) that equals the number (per unit volume) of chains at time *t* that have returned into the active state before instant τ≤t and belong to a meso-domain with activation energy *v*. According to this definition, n(t,t,v) is the number of active chains in meso-domains with activation energy *v* at time *t*. The number of chains that were active at the initial instant t=0 and have not separated from their junctions until time *t* reads n(t,0,v). The number of chains that were active at t=0 and separate from their junctions within the interval [t,t+dt] is given by −∂n/∂t(t,0,v) dt. The number of dangling chains that return into the active state within the interval [τ,τ+dτ] reads P(τ,v)dτ with

(6) $$P\left(\tau ,v\right)=\frac{\partial n}{\partial \tau }\left(t,\tau ,v\right){|}_{t=\tau }.$$

The number of chains (per unit volume) that merged (for the last time) with the network within the interval [τ,τ+dτ] and separate from their junctions within the interval [t,t+dt] is given by $-\partial^{2}n/\partial t\partial \tau \left(t,\tau ,v\right)\;dtd\tau$.

We suppose that the number of active chains (per unit volume) in the meso-domains with various activation energies *v* remain constant,

(7) $$n\left(t,t,v\right)=N_{a}f\left(v\right),$$

where $N_{a}$ is the total number of active chains per unit volume.

The kinetic equations describe the separation of active chains from their junctions (the dissociation of physical bonds) [54,55]

(8) $$\frac{\partial n}{\partial t}\left(t,0,v\right)=-\Gamma \left(v\right)n\left(t,0,v\right),\;\frac{\partial^{2}n}{\partial t\partial \tau }\left(t,\tau ,v\right)=-\Gamma \left(v\right)\frac{\partial n}{\partial \tau }\left(t,\tau ,v\right).$$

Equation (8) implies that the rate of transformation of active chains into the dangling state is proportional to the number of active chains in an appropriate meso-domain. Equation (8) differs from appropriate relations in [56,57], where nonlinear kinetic equations were proposed for the dissociation of physical bonds.

Integrating Equation (8) with initial conditions (6) and (7) with initial conditions (6), we find that

(9) $$n\left(t,0,v\right)=N_{a}f\left(v\right)\exp\left[-\Gamma \left(v\right)t\right],\;\frac{\partial n}{\partial \tau }\left(t,\tau ,v\right)=N_{a}\Gamma \left(v\right)f\left(v\right)\exp\left[-\Gamma \left(v\right)\left(t-\tau \right)\right].$$

Equation (9) differs from the corresponding equations for the rates of dissociation and re-association of the physical bonds that were proposed in [58].

The mechanical energy that is stored in an active chain under shear deformation with small strains is determined by the conventional formula

$$w=\frac{1}{2}\bar{\mu }\epsilon^{2},$$

where $\bar{\mu }$ is the rigidity of a chain and ϵ stands for shear strain. The strain energy density (per unit volume) of the network equals the sum of the mechanical energies of permanent and active temporary chains,

(10) $$W\left(t\right)=\frac{1}{2}\bar{\mu }\left\{N_{p}\epsilon^{2}\left(t\right)+\int_{0}^{\infty }dv\left[n\left(t,0,v\right)\epsilon^{2}\left(t\right)+\int_{0}^{t}\frac{\partial n}{\partial \tau }\left(t,\tau ,v\right){\left(\epsilon (t)-\epsilon (\tau )\right)}^{2}d\tau \right]\right\},$$

where $N_{p}$ stands for the concentration of permanent chains.

The first term that is presented in Equation (10) equals the strain energy of permanent chains. The other term is the strain energy of temporary chains that were active at t=0 and that have not been rearranged within the interval [0,t]. The last term expresses the strain energy of active chains that have last merged with the network at various instants τ∈[0,t]. Equation (10) presumes stresses in dangling chains to relax entirely before these chains merge with the network, which implies that the mechanical energy (at time *t*) that is stored in a chain transformed into the active state at time τ depends on the relative strain $\epsilon^{*}\left(t,\tau \right)=\epsilon \left(t\right)-\epsilon \left(\tau \right)$.

Inserting expression (10) into the Clausius–Duhem inequality

$$-\frac{dW}{dt}+\sigma \frac{d\epsilon }{dt}\geq 0,$$

where σ(t) denotes the shear stress at time *t*, and using Equations (5) and (9), we arrive at the stress–strain relation

(11) $$\sigma \left(t\right)=\mu \left[\epsilon \left(t\right)-\kappa \int_{0}^{\infty }\Gamma \left(v\right)f\left(v\right)dv\int_{0}^{t}\exp\left(-\Gamma (v)(t-\tau )\right)\epsilon \left(\tau \right)d\tau \right]$$

with

$$\mu =\bar{\mu }\left(N_{a}+N_{p}\right),\;\kappa =\frac{N_{a}}{N_{a}+N_{p}}.$$

It follows from Equation (11) that, in a shear oscillatory test with amplitude $\epsilon_{0}$ and angular frequency ω,

$$\epsilon \left(t\right)=\epsilon_{0}\exp\left(ı\omega t\right),$$

the storage, $G^{\prime }\left(\omega \right)$, and loss, $G^{\prime \prime }\left(\omega \right)$, moduli are determined by the equations

(12) $$G^{\prime }\left(\omega \right)=\mu \int_{0}^{\infty }f\left(v\right)\frac{\left(1-\kappa \right)\Gamma^{2}\left(v\right)+\omega^{2}}{\Gamma^{2}\left(v\right)+\omega^{2}}dv,\;G^{\prime \prime }\left(\omega \right)=\mu \int_{0}^{\infty }f\left(v\right)\frac{\kappa \Gamma (v)\omega }{\Gamma^{2}\left(v\right)+\omega^{2}}dv,$$

where Γ(v) is given by Equation (3), and f(v) obeys Equations (4) and (5).

When the rate of dissociation of physical bonds $\Gamma_{0}$ is constant, a combination of Equations (3) and (12) implies that

(13) $$\begin{array}{ccc} G^{\prime }\left(\omega \right) & = & \mu \int_{0}^{\infty }f\left(\ln\frac{\tau }{\tau_{0}}\right)\frac{\left(1-\kappa \right)+\tau^{2}\omega^{2}}{1+\tau^{2}\omega^{2}}d\ln\tau ,\\ G^{\prime \prime }\left(\omega \right) & = & \mu \int_{0}^{\infty }f\left(\ln\frac{\tau }{\tau_{0}}\right)\frac{\kappa \tau \omega }{1+\tau^{2}\omega^{2}}d\ln\tau \end{array}$$

with

$$\tau_{0}=\frac{1}{\Gamma_{0}},\;\tau =\tau_{0}\exp\left(v\right).$$

Equation (13) coincides with Equations (1) and (2) when the function f(v) is given by Equation (4) and κ=1 (the chains in a network are only bridged by physical bonds ). When κ<1 (the chains are bridged by covalent cross-links and physical bonds), Equation (13) provides an extension of the generalized Maxwell model.

To analyze the time-dependent response in the shear relaxation tests with the program

$$\epsilon \left(t\right)=0\;\left(t<0\right),\;\epsilon \left(t\right)=\epsilon_{0}\;\left(t\geq 0\right),$$

we introduce the relaxation modulus $G_{r}\left(t\right)=\sigma \left(t\right)/\epsilon_{0}$ and find, from Equation (11), that

(14) $$G_{r}\left(t\right)=\mu \left[\left(1-\kappa \right)+\kappa \int_{0}^{\infty }f\left(v\right)\exp\left(-\Gamma (v)t\right)dv\right].$$

Equation (14) implies that $G_{r}$ decreases monotonically with time from $G_{r}\left(0\right)=\mu$ to $G_{r}\left(\infty \right)=\mu \left(1-\kappa \right)$.

It follows from Equation (11) that in shear creep tests with the program

$$\sigma \left(t\right)=0\;\left(t<0\right),\;\sigma \left(t\right)=\sigma_{0}\;\left(t\geq 0\right),$$

an increase in the shear strain ϵ with time *t* is governed by the equation

(15) $$\epsilon \left(t\right)=\frac{\sigma_{0}}{\mu }+\kappa \int_{0}^{\infty }f\left(v\right)R\left(t,v\right)dv,$$

where the function

$$R\left(t,v\right)=\Gamma \left(v\right)\int_{0}^{t}\exp\left(-\Gamma (v)(t-\tau )\right)\epsilon \left(\tau \right)d\tau$$

obeys the differential equation

(16) $$\frac{\partial R}{\partial t}\left(t,v\right)=\Gamma \left(v\right)\left(\epsilon (t)-R(t,v)\right),\;R\left(0,v\right)=0.$$

The generalized Maxwell model (1), (2) (to which Equation (12) is reduced at κ=1) cannot reproduce the flattening of the experimental dependencies $G^{\prime \prime }\left(\omega \right)$ at relatively high frequencies ω, as mentioned in the Introduction. To explain why the flattening arises, we suppose that the network consists of entangled chains that were connected by permanent and temporary bonds. When an active chain in transformed into the dangling state, the reptative diffusion of this chain starts [59,60]. It results in the partial disentanglement of this chain and formation of new entanglements. This process stops when the dangling chains return into the active state [61,62].

**Figure 1:**
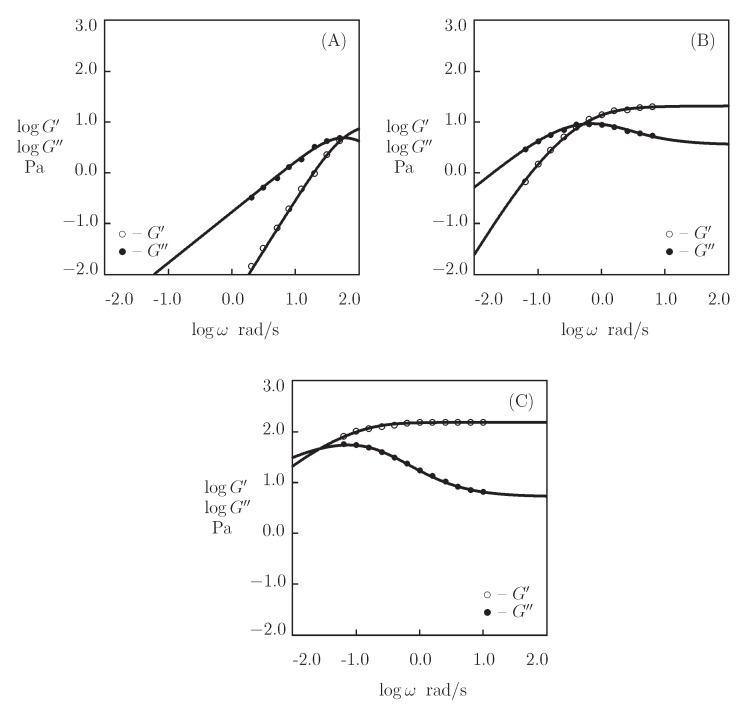
Storage modulus $G^{\prime }$ and loss modulus $G^{\prime \prime }$ versus frequency ω. Symbols: experimental data [67] on hyaluronic acid gel cross-linked by benzoxaborin-saccharide complexation at various pH ((**A**)–pH=6.0, (**B**)–pH=7.4, (**C**)–pH=9.0). Solid lines: the results of simulation.

The viscoelastic response of a gel reflects two kinetic processes at the micro-level: (i) the dissociation and re-association of physical bonds and (ii) disentanglement and re-entanglement of dangling chains. When the volume fraction of water molecules in a gel is large, the rates of disentanglement and re-entanglement of chains are high, and these processes become practically “invisible” in shear oscillatory tests (with ω below 100 rad/s), as their characteristic rates strongly exceed the frequency of oscillations (see Figure 1A, below). The influence of the disentanglement and re-entanglement of chains on the rearrangement of temporary bonds is observed in oscillatory tests as flattening of the graphs $G^{\prime \prime }\left(\omega \right)$ and the formation of high-frequency “tails” induced by interactions between these two processes.

We do not explicitly account for disentanglement and re-entanglement of chains in order to develop a model with a minimal number of adjustable parameters. The effect of this process on the kinetics of rearrangement of physical bonds is phenomenologically described by presuming the rate of their dissociation $\Gamma_{0}$ to increase linearly with frequency of oscillations ω,

(17) $$\Gamma_{0}=\gamma \left(1+K\omega \right),$$

where γ and *K* are material constants. An advantage of Equation (17) is that this assumption does not affect Equations (14) to (16), which characterize the response of hydrogels in “slow” relaxation and creep tests.

Constitutive models with the rate of rearrangement of physical bonds being affected by the intensity of external load are widely used in the analysis of the nonlinear viscoelastic response of solid polymers [63] and polymer melts [64,65]. A similar approach was recently applied in [66] to describe the effect of the frequency of oscillations on the viscoelastic behavior of supramolecular gels.

Equations (3), (4), (12), and (17) provide governing equations for the viscoelastic response of protein-based gels in small-amplitude shear oscillatory tests. These relations involve five adjustable parameters: (i) μ stands for the elastic modulus, (ii) κ is the ratio of the number of physical bonds between chains to the total number of (chemical and physical) cross-links, (iii) Σ is a measure of inhomogeneity of the polymer network, (iv) γ denotes the rate of dissociation of physical bonds, and (v) *K* accounts for the influence of disentanglement and re-entanglement of chains on the rearrangement of temporary bonds.

## 3. Fitting of Experimental Data

The aim of the analysis of observations is twofold: (i) to examine the validity of the assumptions that were used in derivation of the governing equations and to demonstrate the ability of the model to describe experimental data in shear oscillatory tests and relaxation tests on biocompatible supramolecular gels (Section 4), and (ii) to develop structure-property relations in the linear viscoelasticity of protein-based gels that characterize the effects of temperature, concentration, and chemical structure of proteins on material parameters (Section 5).

Adjustable parameters in the governing equations are determined by separately matching each set of experimental data. They are found by the nonlinear regression method to minimize the expression

$$\sum_{\omega }\left[\left(G_{\exp}^{\prime }\left(\omega \right)-G_{\mathrm{sim}}^{\prime }\left(\omega \right)\right){)}^{2}+{\left(G_{\exp}^{\prime \prime }\left(\omega \right)-G_{\mathrm{sim}}^{\prime \prime }\left(\omega \right)\right)}^{2}\right],$$

where summation is performed over all frequencies ω under consideration, $G_{\exp}^{\prime },G_{\exp}^{\prime \prime }$ stand for the storage and loss moduli measured in a test, $G_{\mathrm{sim}}^{\prime },G_{\mathrm{sim}}^{\prime \prime }$ are determined by Equation (12).

## 4. Biocompatible Supramolecular Gels

In order to reveal the ability of the model to describe (and predict in some cases) experimental data, we analyze the observations on biocompatible supramolecular gels that are cross-linked by (i) benzoxaborin-saccharide, (ii) benzaldehyde-thiol, (iii) hydrazine-aldehyde complexes, and by (iv) hydrophobic interactions between fatty acids. Because these gels do not contain covalent cross-links, we set κ=1 in the governing equations, which reduces the number of material parameters to four.

### 4.1. HA Gel Cross-Linked by Benzoxaborin-Saccharide Complexation

We begin with fitting the experimental data on hyaluronic acid (HA) gel that is physically cross-linked by benzoxaborin-saccharide complexation of HA chains end-functionalized with benzoxaborin derivative 2,1-BORIN and fructose (Figueiredo et al. [67]). The Supplementary Material provides the preparation of the gel and experimental conditions. Figure 1 depicts the observations in shear oscillatory tests at room temperature on gels prepared in HEPES buffers with pH=6, 7.4 and 9, where the experimental storage, $G^{\prime }$, and loss, $G^{\prime \prime }$, moduli are plotted versus the angular frequency of oscillations ω together with results of simulation with the material parameters that are listed in Table S1.

Figure 1A demonstrates the viscoelastic behavior of a non-cross-linked gel (because pH=6 is strongly lower than ${\mathrm{pK}}_{a}=8.4$ of 2,1-BORIN, no physical bonds are formed between chains). The gel is characterized by a high rate of disentanglements of chains γ=63 s^−1^ (which implies that the main parts of the graphs $G^{\prime }\left(\omega \right)$ and $G^{\prime \prime }\left(\omega \right)$ are located outside of the conventional interval of frequencies), a low inhomogeneity of the network Σ=0.1, and a small elastic modulus μ=9.8 Pa. Figure 1B,C show that the formation of supramolecular bonds between chains (driven by an increase in pH) leads to a pronounced slowing down of the rearrangement process (γ decreases by three orders of magnitude at $\mathrm{pH}>{\mathrm{pK}}_{a}$), which is accompanied by an increase in the shear modulus μ and the measure of inhomogeneity of the network Σ (by an order of magnitude), and a strong growth of the parameter *K* (reflecting the influence of disentanglements of chains on the kinetics of dissociation of supramolecular complexes).

### 4.2. PEG Gels Cross-Linked by Benzaldehyde-Thiol Complexation

We proceed with fitting observations on two poly(ethylene glycol) (PEG) gels that are cross-linked by benzaldehyde-thiol complexation of tetra-arm PEG chains end-functionalized with benzaldehyde (PEG-BCA) and 4-cyanobenzaldehyde (PEG-CBCA) and tetra-arm PEG chains end-functionalized with thiol (PEG-thiol) (FitzSimon et al. [68]). The Supplementary Material provides details of the preparation procedure and experimental conditions.

**Figure 2:**
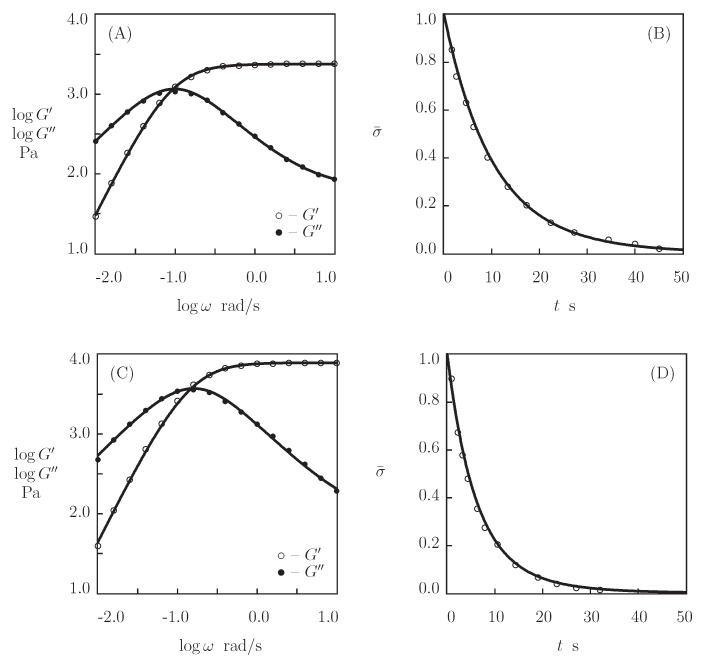
(**A**,**C**)–Storage modulus $G^{\prime }$ and loss modulus $G^{\prime \prime }$ versus frequency ω. (**B**,**D**)–Normalized stress $\bar{\sigma }=\sigma \left(t\right)/\sigma \left(0\right)$ versus relaxation time *t*. Symbols: experimental data [68] on four-arm PEG gels that were cross-linked by benzaldehyde-thiol complexation of PEG-BCA and PEG–thiol chains (**A**,**B**) and PEG-CBCA and PEG-thiol chains (**C**,**D**). Solid lines: the results of simulation (**A**,**C**) and predictions of the model (**B**,**D**).

Figure 2A,C presents the experimental data in shear oscillatory tests on these gels (where $G^{\prime }$ and $G^{\prime }$ are plotted versus frequency ω), and Figure 2B,D reports those in shear relaxation tests (where the relaxation modulus $G_{r}$ is plotted versus relaxation time *t*). To examine the predictive capabilities of the model, we find adjustable parameters in the governing equations (these quantities are collected in Table S2) by matching the experimental data in oscillatory tests (Figure 2A,C), determine $G_{r}\left(t\right)$ from Equation (14), and compare the results of the simulation with experimental data. Figure 2B,D confirms the ability of the model to predict the observations in relaxation tests.

**Figure 3:**
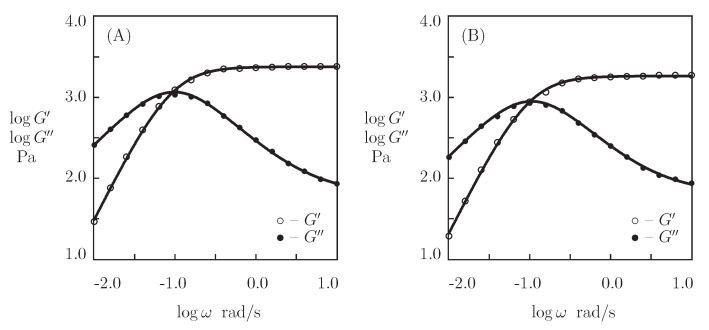
Storage modulus $G^{\prime }$ and loss modulus $G^{\prime \prime }$ versus frequency ω. Symbols: experimental data [68] on four-arm PEG gel cross-linked by benzaldehyde-thiol complexation of PEG-BCA and PEG–thiol chains. (**A**)–virgin sample, (**B**)–cut and healed sample. Solid lines: the results of simulation.

Equation (17) introduces the coefficient *K* to characterize the effect of the disentanglement of chains on the kinetics of rearrangement of supramolecular bonds. According to this definition, the enhancement of the disentanglement process (that is driven by splitting of chains into parts) induces an increase in *K*. To confirm this assertion, two sets of observations in shear oscillatory tests are fitted on PEG gel cross-linked by supramolecular bonds between chains that were end-functionalized with BCA and thiol. Figure 3A plots the observations on a virgin sample and Figure 3B depicts those on a sample cut into pieces and self-healed, together with the results of simulation with the material constants reported in Table S3. This table shows that the scission of chains in the cut-and-healed sample results in the growth of *K* (by 26%) accompanied by the corresponding decrease in the shear modulus μ (by 23%), while the rate of dissociation of bonds γ and the measure of the network inhomogeneity Σ remain unchanged.

### 4.3. HA Gels Cross-Linked by Hydrazine-Aldehyde Complexation

**Figure 4:**
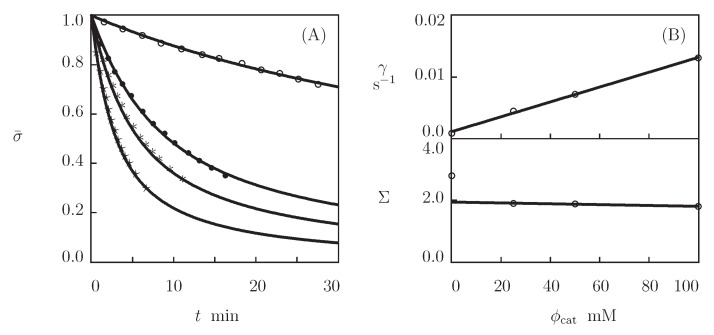
(**A**)–Normalized stress $\bar{\sigma }=\sigma \left(t\right)/\sigma \left(0\right)$ versus relaxation time *t*. Symbols: experimental data [69] on HA gels that were cross-linked by hydrazine-aldehyde complexation with mass fraction of HA ϕ=0.02 and various concentrations of catalyst $\phi_{\mathrm{cat}}$ (◦–$\phi_{\mathrm{cat}}=0$, •–$\phi_{\mathrm{cat}}=25$, ∗–$\phi_{\mathrm{cat}}=50$, ⋆–$\phi_{\mathrm{cat}}=100$ mM). Solid lines: the results of simulation. (**B**)–Parameters γ and Σ versus concentration of catalyst $\phi_{\mathrm{cat}}$. Circles: treatment of observations. Solid lines: the results of simulation.

**Figure 5:**
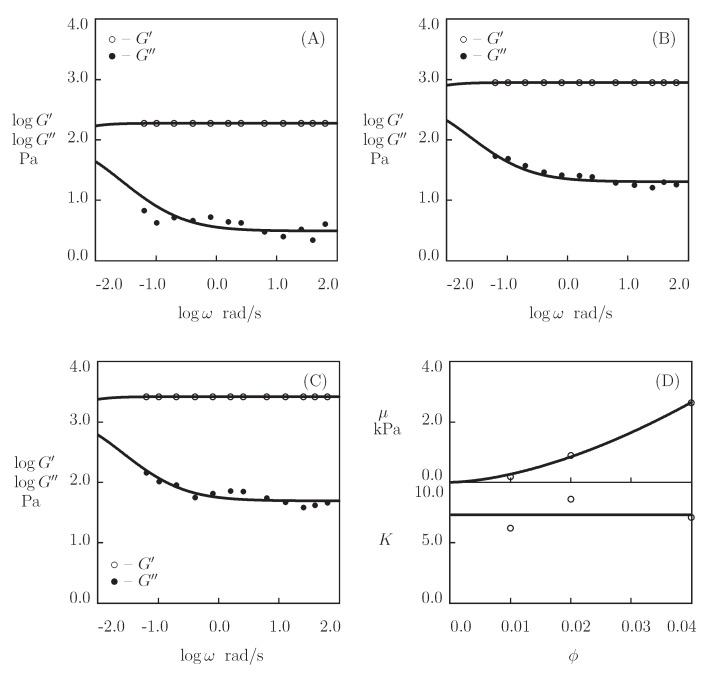
(**A**–**C**)–Storage modulus $G^{\prime }$ and loss modulus $G^{\prime \prime }$ versus frequency ω. Symbols: experimental data [69] on HA gels that were cross-linked by hydrazine-aldehyde complexation with $\phi_{\mathrm{cat}}=50$ mM and various mass fractions of HA ϕ ((**A**)–ϕ=0.01, (**B**)–ϕ=0.02, (**C**)–ϕ=0.04). Solid lines: the results of simulation. (**D**)–Parameters μ and *K* versus mass fraction ϕ of HA. Circles: treatment of observations. Solid lines: the results of simulation.

We now fit the experimental data on hyaluronic acid gels that were physically cross-linked by the complexation of linear HA chains end-functionalized with hydrazine and aldehyde in the presence of 2-(aminomethyl)benzimidazole as a catalyst (Lou et al. [69]). The Supplementary Material describes the preparation of the gels and the experimental conditions. Figure 4A reports the observations in shear relaxation tests on HA gels with various molar fractions of catalyst $\phi_{\mathrm{cat}}$, where the normalized shear stress $\bar{\sigma }\left(t\right)=\sigma \left(t\right)/\sigma \left(0\right)$ is plotted versus the relaxation time *t* (the curves are independent of the concentration of HA chains). Figure 5 presents the experimental data in shear oscillatory tests on the gels with various concentrations of HA ϕ (ranging from 0.01 to 0.04) and a fixed molar fraction of catalyst $\phi_{\mathrm{cat}}=50$ mM.

Figure 4 and Figure 5 show that hydrazine-aldehyde complexes are rather stable (compared with benzaldehyde-thiol complexes), which implies that the moduli $G^{\prime }$ and $G^{\prime \prime }$ are weakly affected by ω in the entire region of frequencies under consideration. We start with matching experimental data in relaxation tests by means of Equation (14) in order to determine adjustable parameters. Keeping in mind that κ=1, we find, from this relation, that

(18) $$\bar{\sigma }\left(t\right)=\int_{0}^{\infty }f\left(v\right)\exp\left(-\Gamma (v)t\right)dv,$$

where Γ(v) and f(v) are given by Equations (3) and (4), respectively. Each set of data in Figure 4A is separately fitted with the help of two parameters, γ and Σ. These quantities are plotted versus the molar fraction of catalyst $\phi_{\mathrm{cat}}$ shown in Figure 4B. The data are approximated by the linear equations

(19) $$\gamma =\gamma_{0}+\gamma_{1}\phi_{\mathrm{cat}},\;\Sigma =\Sigma_{0}+\Sigma_{1}\phi_{\mathrm{cat}},$$

where the coefficients are determined by the least-squares technique. Figure 4B shows that the addition of the catalyst leads to the growth of the rate of dissociation of supramolecular bonds γ, but it does not practically affect the measure of the network inhomogeneity Σ.

We use the values of γ and Σ that were reported in Figure 4B to fit the experimental data in Figure 5. Each set of data shown in Figure 5A–C is approximated by means of two coefficients, μ and *K*, only. Figure 5D illustrates the effect of concentration of HA chains ϕ on these parameters. The data are approximated by the equations

(20) $$\mu =\mu_{1}\phi^{m},\;K=K_{0}$$

with the coefficients being calculated by the least-squares technique ($\mu_{1}$ and *m* are listed in Table S4).

### 4.4. PEG-DDI Gel with Hydrophobic Interactions between Fatty Acids

We study the observations on a gel prepared by the copolymerization of linear PEG chains with dimer fatty acid-based diisocyanate (DDI) to demonstrate the applicability of the above algorithm of fitting observations to hydrogels with other types of supramolecular bonds (Mihajlovic et al. [70]). The Supplementary Material provides a description of the prepation procedure for the PEG-DDI gel and experimental conditions.

**Figure 6:**
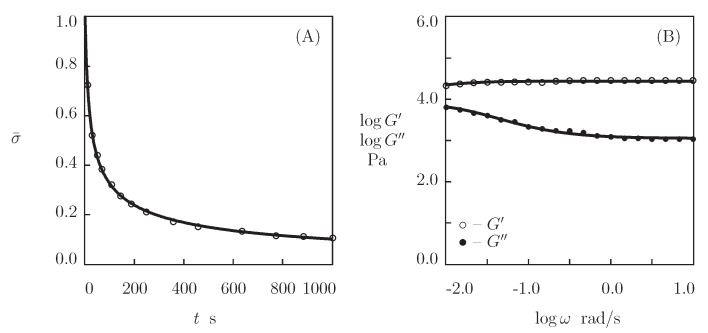
(**A**)–Normalized stress $\bar{\sigma }=\sigma \left(t\right)/\sigma \left(0\right)$ versus relaxation time *t*. (**B**)–Storage modulus $G^{\prime }$ and loss modulus $G^{\prime \prime }$ versus frequency ω. Circles: experimental data [70] on PEG-DDI gel with hydrophobic interactions. Solid lines: the results of simulation.

First, the observations in a shear relaxation test (as in Figure 6A) are matched by means of two adjustable parameters, γ and Σ. Afterwards, we approximate the experimental data in a shear oscillatory test (Figure 6B) with the help of the other two parameters, μ and *K*. Figure 6 reveals good agreement between the experimental data and the results of simulation with the material constants collected in Table S5.

### 4.5. Discussion

Figure 2, Figure 3, Figure 4, Figure 5 and Figure 6 demonstrate the ability of the model (with only four adjustable parameters) to describe the experimental data in shear relaxation tests and small-amplitude oscillatory tests (these sets of observations for $G_{r}\left(t\right)$, $G^{\prime }\left(\omega \right)$ and $G^{\prime \prime }\left(\omega \right)$) simultaneously on gels with various types of supramolecular bonds (benzaldehyde-thiol complexation, hydrazine-aldehyde complexation, and hydrophobic interactions).

When the rate of dissociation of bonds γ is relatively large, the fitting of observations in shear oscillatory tests allows the viscoelastic response of supramolecular gels in relaxation tests to be predicted (Figure 2B,D). Material parameters in the governing equations are determined by matching observations in relaxation and oscillatory tests when this coefficient is small (which implies that the storage and loss moduli become practically independent of frequency) (Figure 4, Figure 5 and Figure 6).

The experimental data under consideration cannot be adequately described by means of the generalized Maxwell model (1), (2) as they reveal a pronounced flattening of the curves $G^{\prime \prime }\left(\omega \right)$ at relatively high frequencies. An advantage of the proposed model is that it ensures good agreement with observations due to the presence of the coefficient *K* accounting for the effect of disentanglement and re-entanglement of chains on the rearrangement of supramolecular bonds. When the flattening of experimental diagrams $G^{\prime \prime }\left(\omega \right)$ is relatively weak (Figure 2C), the coefficient *K* remains small (below 0.1). However, it increases up to unity (Table S5) and even higher (Figure 5D) when the flattening of the curve $G^{\prime \prime }\left(\omega \right)$ dominates the viscoelastic response.

The physical meaning of the coefficient *K* is clarified by the observations that are reported in Figure 1 and Figure 3. Figure 1 reveals that the pH-driven formation of supramolecular bonds between chains (that induces a strong deceleration of the disentanglement process) results in a substantial growth of the parameter *K* (as the rates of disentanglement of chains and rearrangement of physical bonds become comparable). A comparison of the observations on virgin and self-healed samples shown in Figure 3 shows that the cutting of chains into pieces (which enhances the disentanglement process) induces an appropriate increase in the coefficient *K*.

## 5. Protein-Based Gels

Our aim is twofold: (i) to demonstrate the ability of the model to fit the experimental data on protein- and peptide-based gels, and (ii) to examine how the viscoelastic behavior of these gels is affected by their composition and chemical structure (amino acid sequences), the concentration of polymer in pre-gel solutions, and temperature.

### 5.1. Peptide-Functionalized PEG Gel

We begin with fitting the experimental data on tetra-arm PEG gel end-functionalized with acidic (A${}_{4H3}$) and basic (B${}_{4H3}$) peptides and cross-linked by coiled coil complexes between the peptides (Tunn et al. [71]). The Supplementary Material describes the preparation of the gel and experimental conditions.

**Figure 7:**
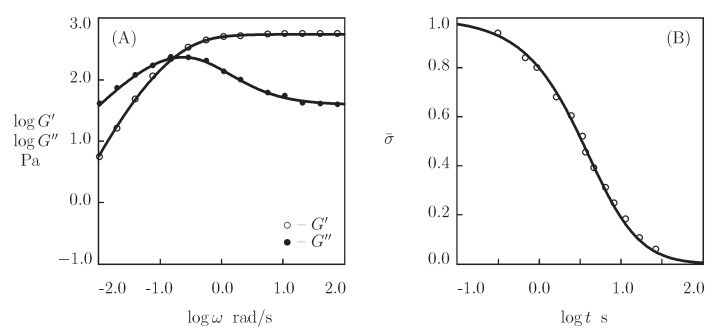
(**A**)–Storage modulus $G^{\prime }$ and loss modulus $G^{\prime \prime }$ versus frequency ω. Symbols: experimental data [71] on peptide-functionalized PEG gel. Solid lines: results of simulation. (**B**)–Normalized stress $\bar{\sigma }=\sigma \left(t\right)/\sigma \left(0\right)$ versus relaxation time *t*. Circles: experimental data. Solid line: the prediction of the model.

Observations in shear oscillatory test and shear relaxation test are reported in Figure 7, together with results of the simulation with the material constants collected in Table S6. As the normalized shear stress $\bar{\sigma }$ approaches zero in relaxation test (Figure 7B), we set κ=1. The remaining parameters (μ, γ, Σ, and *K*) are found by matching the observations in the oscillatory test (Figure 7A). These quantities are used to calculate the decay in stress with time in the relaxation test by means of Equation (18). Figure 7B shows good agreement between the predictions of the model and experimental data.

### 5.2. Protein-Functionalized PEG Gels

We proceed with matching the experimental data on tetra-arm PEG gels that were end-functionalized with three recombinant proteins (EPE, L37I, and L37V) and cross-linked by coiled coil complexes between the midblock domains of the proteins (Dooling and Tirrell [47]). The EPE proteins with a triblock architecture contain endblocks E (elastin-like polypeptides), a helical midblock domain P (derived from the cartilage oligomeric matrix protein), and N- and C-terminal cysteine residues. EPE, L37I, and L37V proteins differ from one another by a single amino acid residue that was located at position 37 in the midblock. The Supplementary Material provides a description of the recombinant proteins, preparation of the gels, and experimental conditions.

**Figure 8:**
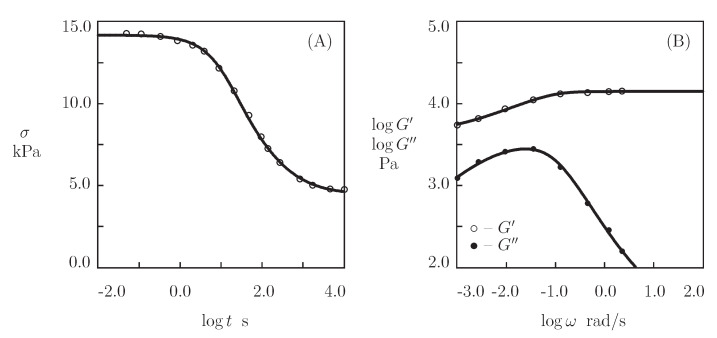
(**A**)–Shear stress σ versus relaxation time *t*. (**B**)–Storage modulus $G^{\prime }$ and loss modulus $G^{\prime \prime }$ versus frequency ω. Symbols: experimental data [47] on PEG-EPE gel. Solid lines: the results of simulation.

**Figure 9:**
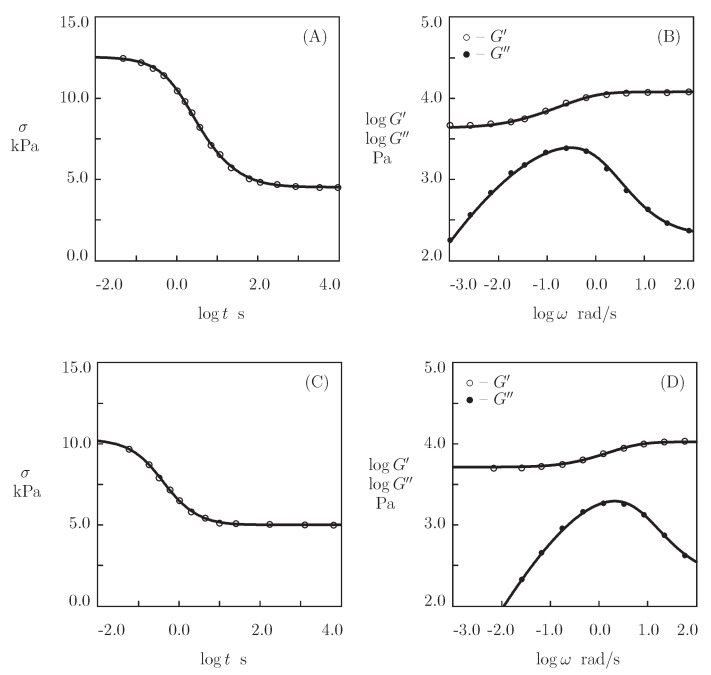
(**A**,**C**)–Shear stress σ versus relaxation time *t*. (**B**,**D**)–Storage modulus $G^{\prime }$ and loss modulus $G^{\prime \prime }$ versus frequency ω. Symbols: experimental data [47] on PEG-L37I (**A**,**B**) and PEG-L37V (**C**,**D**) gels. Solid lines: the results of simulation.

Figure 8 and Figure 9, together with results of simulation with the material constants listed in Table S7, reports the observations in shear relaxation tests and shear oscillatory tests on PEG-EPE, PEG-L37I, and PEG-L37V gels. A characteristic feature of these gels is that the shear stresses σ do not vanish with time in relaxation tests, but they approach some ultimate values (which means that the chains in the polymer network are connected by temporary and permanent cross-links). For each material under consideration, parameters γ, Σ, and κ are determined by an approximation of the experimental data in relaxation tests. Afterwards, the coefficients μ and *K* are found by matching the observations in oscillatory tests.

Table S7 shows that amino acid sequences in the midblocks of proteins strongly affect the mechanical properties of these gels. Changes in a single amino acid residue induce an increase in the rate of dissociation of coiled coils γ by two orders of magnitude (from 0.11 to 6.1 s^−1^). These changes are accompanied by a reduction in the shear modulus μ by 25%, a decrease in Σ by 35%, and a decay in *K* by a factor of 7.

### 5.3. The Effect of Chemical Structure

In order to evaluate the influence of amino acid sequences on the viscoelastic response of protein gels, two sets of experimental data are fitted on telechelic gels, PC_10_P and PC_30_P, which are physically cross-linked by coiled coil complexes (Olsen et al. [72]). The gels were constructed from two helical end blocks (P) that were linked by 10 or 30 repeats of the nonapeptide sequence (C) served as flexible polyanionic linkers. The Supplementary Material describes the structure of recombinant proteins PC_10_P and PC_30_P, the preparation procedure for the gels, and the experimental conditions.

**Figure 10:**
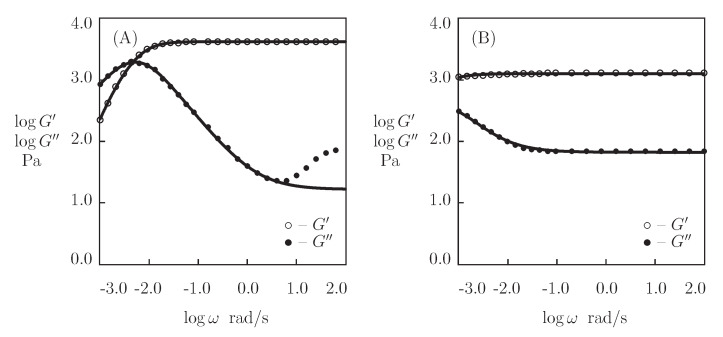
Storage modulus $G^{\prime }$ and loss modulus $G^{\prime \prime }$ versus frequency ω. Symbols: experimental data [72] on protein gels PC${}_{10}$P (**A**) and PC${}_{30}$P (**B**). Solid lines: the results of simulation.

Figure 10 reports the experimental data in shear oscillatory tests on these gels, together with results of simulation with the material parameters collected in Table S8. In the approximation procedure, we set κ=1 and fit each set of observations separately with the help of four adjustable parameters, μ, γ, Σ, and *K*. Although Figure 10 reveals reasonable agreement between the observations and their description by the model, some deviations should be mentioned between the results of numerical analysis for PC_10_P gel and the data on $G^{\prime \prime }$ at high frequencies ω.

Table S8 shows that an increase in the number of repeats in the sequence C results in a noticeable decrease in the elastic modulus μ (by a factor of 3) and a rate of dissociation of coiled coil complexes γ (by a factor of 9), an increase in the measure of inhomogeneity of the network Σ (by a factor of 3), and a pronounced (by two orders of magnitude) growth of the coefficient *K*.

### 5.4. The Effect of Concentration

Two sets of observations are analyzed to assess the influence of concentration of proteins in pre-gel solutions on the viscoelastic response of protein gels.

First, the experimental data are matched on protein P_4_ gels that are cross-linked by coiled coil complexes (Glassman et al. [73] and Tang et al. [74,75]). Recombinant protein P_4_, with the structure C_10_(PC_10_)_4_, consists of four coiled-coil self-associating domains P on the protein backbone joined by flexible polyelectrolyte linkers C_10_. The Supplementary Material describes the preparation procedure for the gels and experimental conditions.

**Figure 11:**
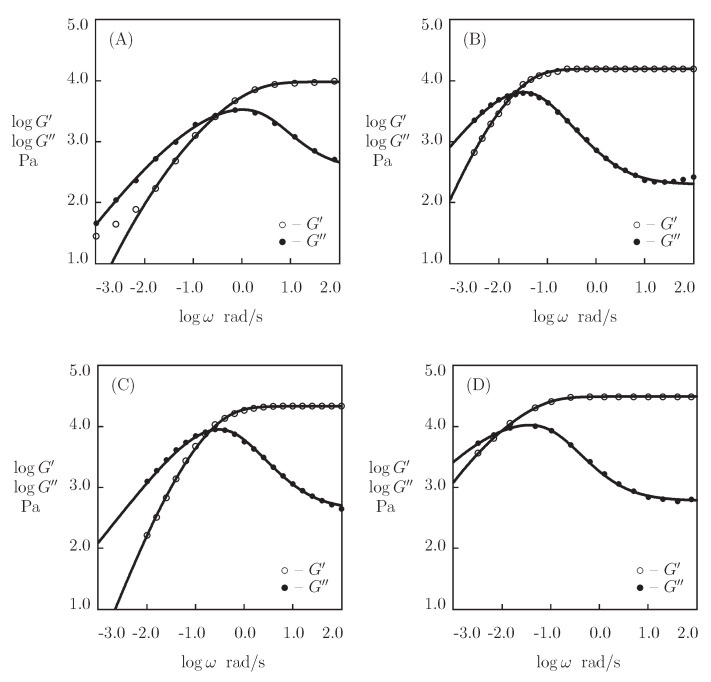
Storage modulus $G^{\prime }$ and loss modulus $G^{\prime \prime }$ versus frequency ω. Symbols: experimental data on P${}_{4}$ gels with various mass fractions of proteins proteins ϕ (**A**)–ϕ=0.15 [74], (**B**)–ϕ=0.22 [73], (**C**)–ϕ=0.25 [75], (**D**)–ϕ=0.30 [73]. Solid lines: the results of simulation.

Figure 11 presents the eperimental data in shear oscillatory tests on P_4_ gels with various concentrations of proteins ϕ (ranging from 0.15 to 0.30). The data are plotted together with the results of simulation with the material parameters reported in Figure 13. In order to reduce the number of adjustable parameters, we suppose that κ=1 and fit each set of observations in Figure 11 with the help of four parameters (μ, γ, Σ, and *K*).

For comparison, the experimental data are approximated on protein CCK-CCE gels that were prepared by mixing two proteins, CCE-(GB_1_)_4_-CCE and CCK-(GB_1_)_5_-CCK-(GB_1_)_5_-CCK (with the same recombinant globular protein GB_1_), phycally cross-linked by hetero-coiled-coil complexes that formed by CCK and CCE polypeptide blocks (Sun et al. [76]). The Supplementary Material provides a description of the preparation procedure for the gels and experimental conditions.

**Figure 12:**
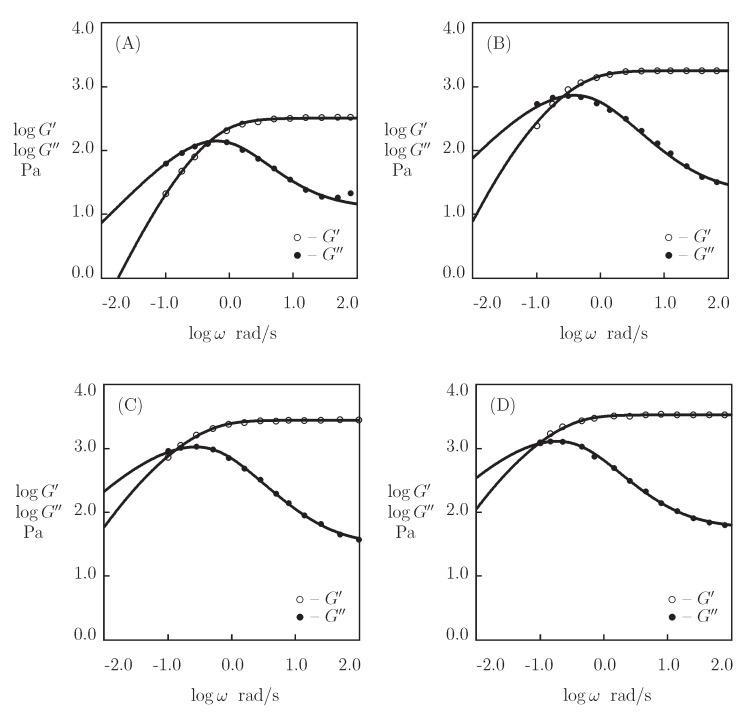
Storage modulus $G^{\prime }$ and loss modulus $G^{\prime \prime }$ versus frequency ω. Symbols: experimental data [76] on CCK-CCE gels with various concentrations of proteins ϕ ((**A**)–ϕ=0.04, (**B**)–ϕ=0.10, (**C**)–ϕ=0.13, (**D**)–ϕ=0.16)). Solid lines: the results of simulation.

**Figure 13:**
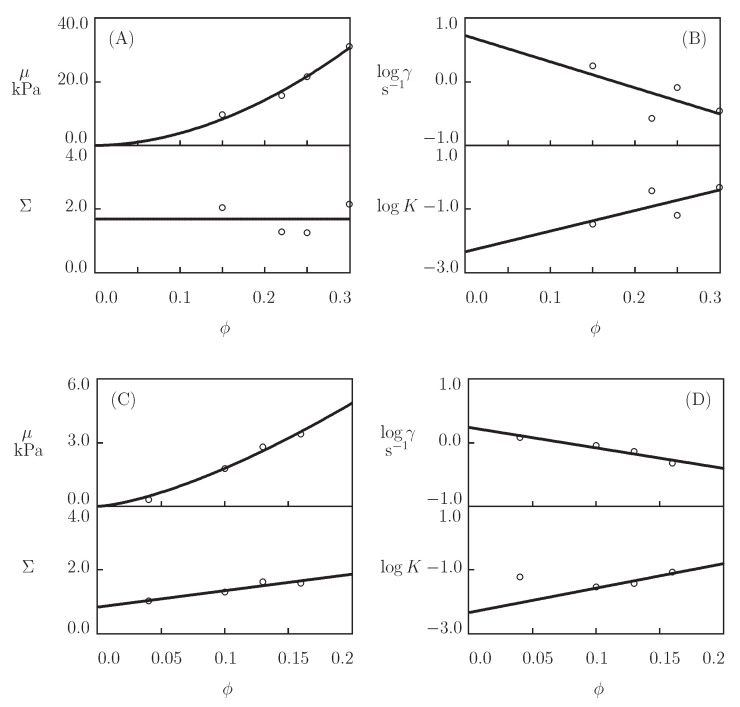
Parameters μ, Σ (**A**,**C**) and γ, *K* (**B**,**D**) versus concentration of proteins ϕ. Circles: treatment of observations on P${}_{4}$ (**A**,**B**) and CCK-CCE (**C**,**D**) gels. Solid lines: the results of simulation.

Figure 12 presents observations in shear oscillatory tests on CCK-CCE gels with various concentrations of proteins in pre-gel solutions ϕ (ranging from 0.04 to 0.16). Each set of data is matched separately with the help of four parameters (μ, γ, Σ, and *K*). Figure 13 illustrates the changes of these quantities with ϕ. The data are approximated by the phenomenological equations

(21) $$\mu =\mu_{1}\phi^{m},\;\Sigma =\Sigma_{0}+\Sigma_{1}\phi ,\;\log\gamma =\gamma_{0}+\gamma_{1}\phi ,\;\log K=K_{0}+K_{1}\phi$$

with the coefficients being calculated by the least-squares technique (parameters $\mu_{1}$ and *m* are collected in Table S4).

Figure 13 and Table S4 show that the elastic modulus μ increases strongly with the concentration of proteins ϕ. This growth is in accordance with the observations on HA gels cross-linked by hydrazine-aldehyde complexation (Figure 5D). Unlike the HA gels, for which the rate of dissociation of supramolecular bonds γ remains independent of polymer concentration, Figure 13B,D demonstrates a pronounced (by an order of magnitude) reduction in γ that is accompanied by a noticeable growth of the coefficient *K*.

For all of the gels under consideration, the exponent *m* presented in Equations (20) and (21) adopts similar values belonging to the interval between 1.4 and 1.9. These values are slightly lower than the m=2.3 predicted by the Rubinstein–Semenov theory [77] and conventionally employed in the analysis of the viscoelastic response of supramolecular gels [78].

### 5.5. The Effect of Temperature

The effect of temperature *T* on the mechanical properties of synthetic supramolecular gels was recently reviewed in [79]. Three sets of observations are analyzed to examine the characteristic features of the thermo-viscoelastic behavior of protein gels.

**Figure 14:**
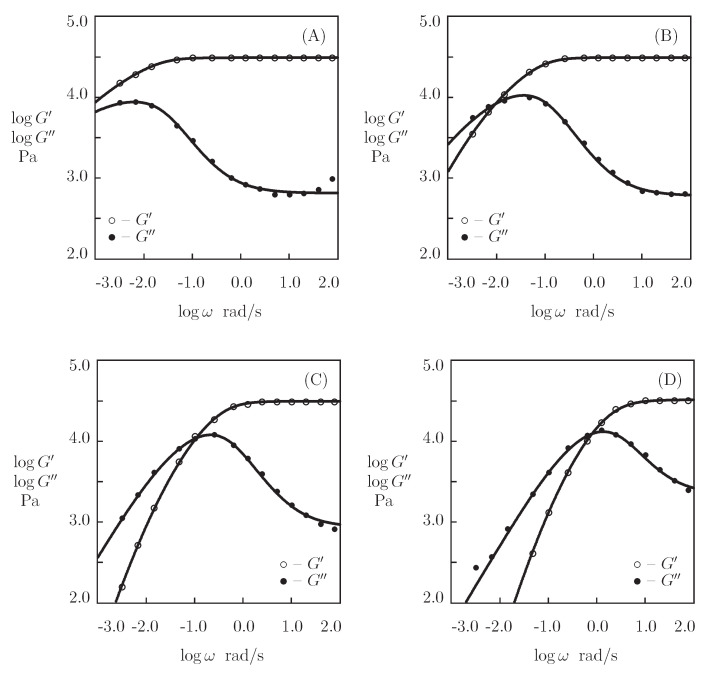
Storage modulus $G^{\prime }$ and loss modulus $G^{\prime \prime }$ versus frequency ω. Symbols: experimental data [73] on P${}_{4}$ protein gel at various temperatures *T* ((**A**)–T=5, (**B**)–T=20, (**C**)–T=35, (**D**)–T=50${}^{\circ }$C). Solid lines: the results of simulation.

We begin with fitting the observations in shear oscillatory tests (Figure 14) on protein P_4_ gel with a fixed concentration of proteins ϕ=0.3 at temperatures T=5, 20, 35, and 50 °C (Glassman et al. [73]). Figure 11 presents the experimental data on P_4_ gels with various concentrations of proteins ϕ at room temperature.

**Figure 15:**
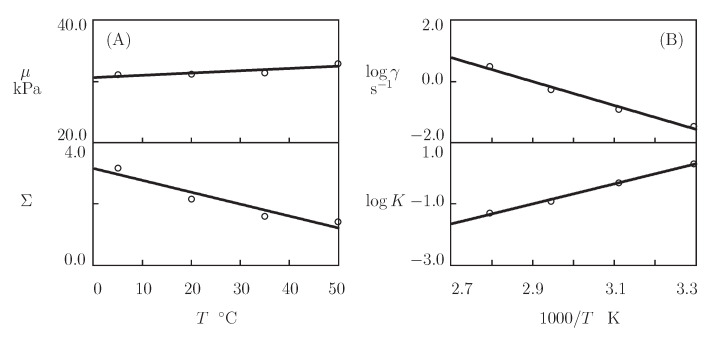
Parameters μ, Σ (**A**) and γ, *K* (**B**) versus temperature *T*. Circles: treatment of observations on P${}_{4}$ gel. Solid lines: the results of simulation.

Presuming κ=1, we separately match each set of observations in Figure 14 by means of four parameters (μ, Σ, γ, and *K*). Their best-fit values are plotted versus temperature *T* in Figure 15. The evolution of the coefficients μ and Σ with *T* (Figure 15A) is described by the linear equations

(22) $$\mu =\mu_{0}+\mu_{1}T,\;\Sigma =\Sigma_{0}+\Sigma_{1}T,$$

where *T* is measured in °C, and the coefficients are found by the least-squares technique. Figure 15B illustrates the effect of temperature on γ and *K*. The data are approximated by the Arrhenius dependencies

(23) $$\log\gamma =\gamma_{0}-\frac{\gamma_{1}}{T},\;\log K=K_{0}+\frac{K_{1}}{T},$$

where *T* stands for the absolute temperature. The coefficients shown in Equation (23) are calculated by the least-squares method. The corresponding activation energies are given by

$$E_{\mathrm{afl}}=\gamma_{1}R\ln10,\;E_{\mathrm{aK}}=K_{1}R\ln10,$$

where R denotes the universal gas constant.

Figure 15A reveals that μ increases slightly, whereas Σ decreases noticeably with *T* (which means that the polymer network becomes more homogeneous when the temperature grows). Figure 15B shows that the rate of dissociation of bonds γ increases, while the parameter *K* (that accounts for the influence of disentanglement of chains on rearrangement of coiled coil complexes) decreases with *T*. Equation (23) adequately describes changes in these parameters with similar activation energies $E_{\mathrm{afl}}$ and $E_{\mathrm{aK}}$ (as seen in Table S9).

We proceed with matching the observations on protein o-Cys-P_4_-Cys gels with two concentrations of proteins in pre-gel solutions (Tang et al. [74]). Protein Cys-P_4_-Cys is a modification of the P_4_ protein achieved by coupling the cysteine residues near the N- and C-termini. Protein o-Cys-P_4_-Cys was prepared by the oxidation of Cys-P_4_-Cys in a buffer solution. The Supplementary Material provides details of the synthesis of gels and the experimental conditions.

**Figure 16:**
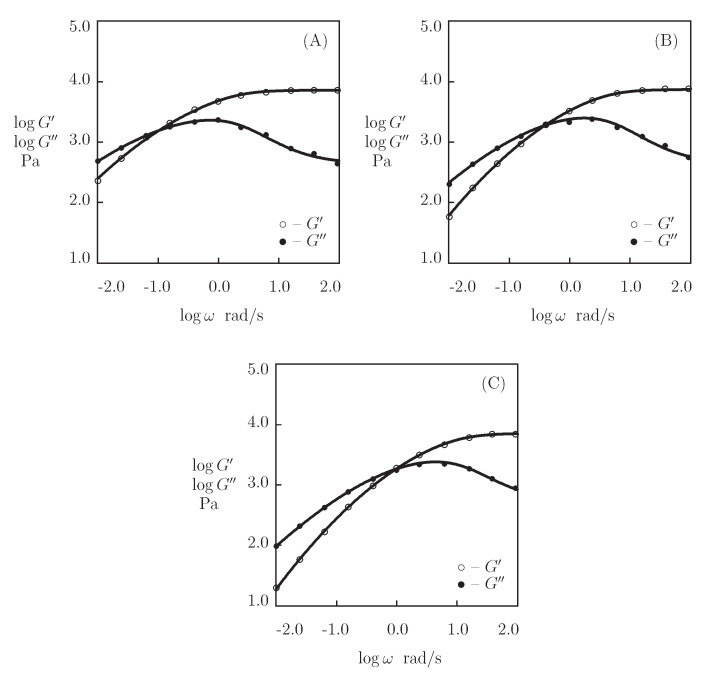
Storage modulus $G^{\prime }$ and loss modulus $G^{\prime \prime }$ versus frequency ω. Symbols: experimental data [74] on o-Cys-P${}_{4}$-Cys gel (mass fraction of proteins 0.1) at various temperatures *T* ((**A**)–T=15, (**B**)–T=25, (**C**)–T=35${}^{\circ }$C). Solid lines: the results of simulation.

**Figure 17:**
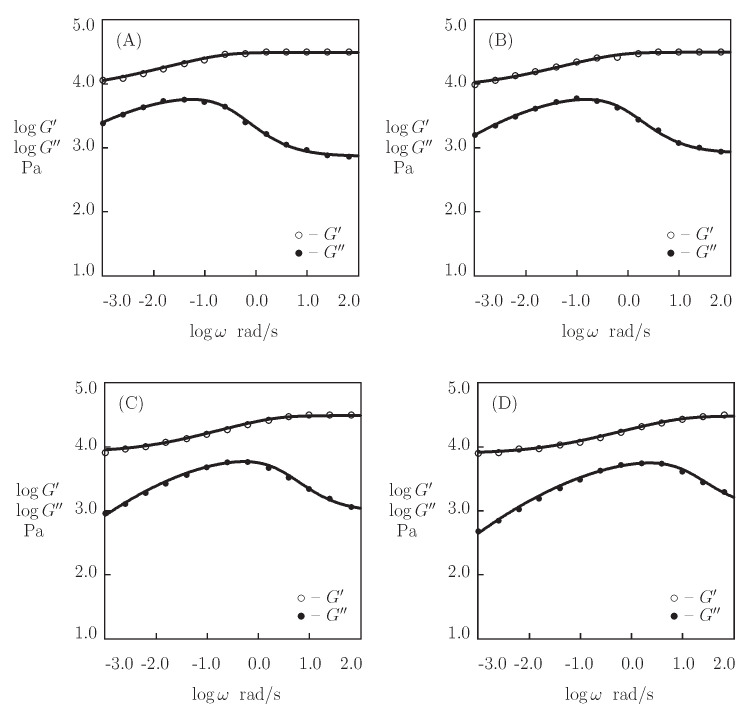
Storage modulus $G^{\prime }$ and loss modulus $G^{\prime \prime }$ versus frequency ω. Symbols: experimental data [74] on o-Cys-P${}_{4}$-Cys gel (mass fraction of proteins 0.2) at various temperatures *T* ((**A**)–T=15, (**B**)–T=25, (**C**)–T=35, (**D**)–T=45${}^{\circ }$C). Solid lines: the results of simulation.

**Figure 18:**
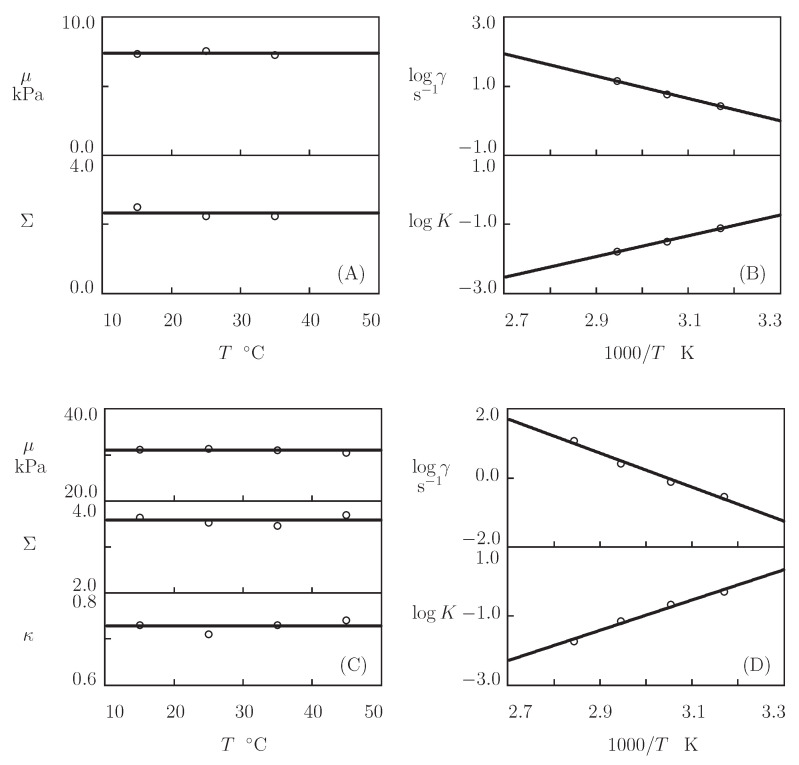
Parameters μ, Σ, κ (**A**,**C**) and γ, *K* (**B**,**D**) versus temperature *T*. Circles: treatment of observations [74] on o-Cys-P${}_{4}$-Cys gels with mass fractions of proteins 0.1 (**A**,**B**) and 0.2 (**C**,**D**). Solid lines: the results of simulation.

The experimental data on o-Cys-P_4_-Cys gels with a concentrations of proteins in pre-gel solutions ϕ=0.1 and 0.2 are reported in Figure 16 (at temperatures T=15, 25, and 35 °C) and Figure 17 (at temperatures T=15, 25, 35, and 45 °C), respectively. Each set of data is separately fitted by the model with five adjustable parameters. The results of numerical analysis show that κ=1 when ϕ=0.1 (Figure 16), but it differs from unity at higher concentration of proteins ϕ=0.2 (Figure 17). The best-fit values of the coefficients μ, Σ, κ, γ, and *K* are plotted versus temperature in Figure 18. The data shown in Figure 18A,C are approximated by temperature-independent constants,

(24) $$\mu =\mu_{0},\;\Sigma =\Sigma_{0},\;\kappa =\kappa_{0}.$$

The data shown in Figure 18B,D are fitted with the Arrhenius dependencies (23). Table S10 collects the corresponding activation energies $E_{\mathrm{afl}}$ and $E_{\mathrm{aK}}$.

Figure 18A,C shows that the elastic modulus μ (characterizing the concentration of cross-links between chains), the coefficient κ (the ratio of the number of temporary bonds to the total number of covalent and non-covalent cross-links), and the parameter Σ (characterizing the distribution of energies for the rearrangement of physical bonds) of o-Cys-P_4_-Cys gels are independent of temperature. However, these quantities are strongly affected by the concentration of proteins ϕ. An increase in ϕ induces: (i) a pronounced growth of the elastic modulus μ (in accordance with the observations reported in Figure 13), (ii) transformation of some temporary bonds into permanent cross-links (due to the formation of aggregates whose dissociation energy strongly exceeds the energy of thermal fluctuations at all temperatures *T* under consideration), and (iii) a noticeable growth of inhomogeneity of the network Σ.

The conclusion that the elastic modulus μ of o-Cys-P_4_-Cys gels remains practically independent of *T* (Figure 18A,C) is in accordance with the results that are depicted in Figure 15A for P_4_ gel. However, the fact that Σ of o-Cys-P_4_-Cys gels is not affected by temperature contradicts the conclusion drawn from fitting the observations on P_4_ gel. This difference in the thermo-mechanical response may be attributed to the stabilizing effect of the cysteine residues in the Cys-P_4_-Cys proteins.

Figure 18B,D and Table S10 reveal that the effect of temperature *T* on the rate of dissociation of the physical bonds γ and the coefficient *K* (reflecting the influence of disentanglement and re-entanglement of chains on the rearrangement of coiled coil complexes) is described by the Arrhenius laws (23) with similar activation energies $E_{\mathrm{afl}}$ and $E_{\mathrm{aK}}$.

Table S10 demonstrates that the coefficients $E_{\mathrm{afl}}$ substantially increase with the concentration of proteins ϕ in the gels. A comparison of the data that are shown in Tables S9 and S10 shows that the presence of oxidized cysteine residues induces a strong increase in the activation energies.

The closeness of the $E_{\mathrm{afl}}$ and $E_{\mathrm{aK}}$ values (Tables S9 and S10) may be explained, as follows. When *T* changes in the (rather narrow) physiological interval of temperatures, its growth leads to an increase in the rate of dissociation of temporary bonds, while does not substantially affect the rate of disentanglement of chains. At low temperatures *T* (small γ), changes in the network structure that are driven by the disentanglement and re-entanglement of chains have sufficient time to accumulate between subsequent dissociation events and affect the rearrangement of supramolecular bonds. At higher temperatures (large γ), their influence on the rearrangement process weakens as changes in the network structure between subsequent dissociation events become negligible.

### 5.6. Discussion

Figure 7, Figure 8, Figure 9, Figure 10, Figure 11 and Figure 12, Figure 14, Figure 16, and Figure 17 demonstrate that the model adequately describes the experimental data in small-amplitude shear oscillatory tests and shear relaxation tests on peptide- and protein-modified PEG gels (Figure 7 and Figure 8) and protein-based gels with various modifications of the chemical structure of proteins (Figure 8, Figure 9 and Figure 10) and various concentrations (Figure 11 and Figure 12) in the physiological interval of temperatures *T* (Figure 14, Figure 16, and Figure 17).

Figure 8, Figure 9, and Figure 17 reveal the characteristic feature of protein-based gels: the development of strong coiled coil complexes whose activation energy for dissociation noticeably exceeds the energy of thermal fluctuations in the interval of physiological temperatures (physical bonds that formed by these complexes are treated as permanent in the model). The presence of strong (permanent) bonds between chains is observed in experiments as a decay in stress in shear relaxation tests to non-zero values (Figure 8 and Figure 9) and the formation of low-frequency plateaus on the graphs $G^{\prime }\left(\omega \right)$ in shear oscillatory tests (Figure 17).

An analysis of the effects of the concentration of proteins ϕ and temperature *T* on the viscoelastic behavior of protein-based gels leads to the following conclusions:

1. Figure 13A and Figure 18A,C show that an increase in the concentration of proteins in pre-gel solutions ϕ induces a strong increase in the elastic modulus μ described by Equations (20) and (21) with the exponent *m* ranging from 1.4 to 1.9. This increase is accompanied by a linear growth of heterogeneity of the network (that is characterized by the coefficient Σ).
2. Figure 13B and Figure 18B,D demonstrate that the growth of ϕ induces a pronounced decrease in the rate of dissociation of coiled coil complexes γ and an increase in the coefficient *K* (that reflects the influence of disentanglement of chains on the rearrangement of physical bonds). These changes (as described by Equation (21)) may be explained by the strengthening of interactions between coiled coil complexes that are located in close vicinity of each other (these interactions slow down the rearrangement process) and the enhancement of the effect of disentanglement caused by the growth of the effect of disentanglement of concentration of chains.
3. Figure 15A and Figure 18A,C reveal that an increase in temperature *T* does not substantially affect the elastic modulus μ (that is treated as a measure of the concentration of physical bonds between chains), but it can induce a pronounced growth of their strength (that is modeled as a transformation of some temporary bonds into permanent), accompanied by a decrease in the measure of inhomogeneity of the polymer network Σ.
4. Figure 15B and Figure 18B,D show that these changes in the network structure occur together with an increase in the rate of dissociation of bonds γ and a reduction in the coefficient *K*. Arrhenius Equations (23) with similar values of the activation energies $E_{\mathrm{afl}}$ and $E_{\mathrm{aK}}$ describe the effect of temperature on γ and *K*.

## 6. Conclusions

A simple model is derived for the linear viscoelastic behavior of protein-based gels. Chains in the polymer network being bridged by physical (temporary) bonds between coiled coil complexes of protein blocks and permanent (covalent) cross-links between functional groups of amino acids is a characteristic feature of these materials (that distinguishes their response from that of synthetic supramolecular gels).

An advantage of the model is that it only involves five material parameters with transparent physical meaning. This allows structure-property relations to be developed in order to characterize the effects of composition (amino acid sequences and protein motifs), concentration of proteins, and temperature on the viscoelastic response of protein-based gels. Three parameters in the model are conventional: the elastic modulus of the polymer network μ, the rate of dissociation of temporary bonds γ, and the measure of inhomogeneity of the network Σ. The coefficient κ equals the ratio of the number of transient bonds to the total number of temporary and permanent cross-links between chains. The coefficient *K* reflects the influence of disentanglement and re-entanglement of chains on the rate of dissociation of physical bonds.

The model is applied to describe the observations in shear relaxation tests and small-amplitude oscillatory tests on (i) functionalized hyaluronic acid (HA) gels that are cross-linked by benzoxaborin-saccharide and hydrazine-aldehyde complexes, (ii) functionalized multi-arm poly(ethylene glycol) (PEG) gels that are cross-linked by benzaldehyde-thiol complexes, hydrophobic interactions between fatty acids, and associating coiled coil bonds between peptides, and (iii) several recombinant protein gels (P_4_, PC_10_P, PC_30_P, o-Cys-P_4_-Cys, and CCK-CCE) with temporary and permanent cross-links.

Figure 2 and Figure 4, Figure 5, Figure 6, Figure 7, Figure 8 and Figure 9 show good agreement between the results of simulation and the experimental data in shear relaxation and oscillatory tests (which means that the model with five adjustable coefficients adequately describes three sets of observations for σ(t), $G^{\prime }\left(\omega \right)$, and $G^{\prime \prime }\left(\omega \right)$ simultaneously). Figure 4B, Figure 5D, and Figure 11, Figure 12, Figure 13, Figure 14, Figure 15, Figure 16, Figure 17 and Figure 18 confirm that the material parameters evolve consistently with the temperature *T* and concentrations of polymers ϕ and catalysts $\phi_{\mathrm{cat}}$ in pre-gel solutions. Section 4.5 and Section 5.6 provide a detailed discussion of their influence on the viscoelastic response of protein-based gels.

## Data Availability

Not applicable.

## Supplementary Materials

The following are available online at https://www.mdpi.com/article/10.3390/bioengineering8060073/s1, S1: Chemical structure and preparation of hydrogels, Table S1: Material parameters for HA gel cross-linked by benzoxaborin-saccharide complexation at various pH (Figure 1), Table S2: Material parameters for HA gel cross-linked by benzoxaborin-saccharide complexation at various pH (Figure 2), Table S3: Material parameters for virgin and healed PEG gels cross-linked by benzaldehyde-thiol complexation (Figure 3), Table S4: Parameters $\mu_{1}$ and *m* in Equations (20) and (21), Table S5: Material parameters for PEG-DDI gel (Figure 6), Table S6: Material parameters for peptide-functionalized PEG gel (Figure 7), Table S7: Material parameters for protein-functionalized PEG gels (Figure 8 and Figure 9), Table S8: Material parameters for telechelic protein gels (Figure 10), Table S9: Activation energies $E_{\mathrm{afl}}$ and $E_{\mathrm{aK}}$ for P_4_ protein gel (Figure 15), Table S10: Activation energies $E_{\mathrm{afl}}$ and $E_{\mathrm{aK}}$ for o-Cys-P_4_-Cys protein gels (Figure 18).
