# Supplementary material for: Thermo-Viscoelastic Response of Protein-Based Hydrogels
Source: Bioengineering (Basel). 2021 May 31;8(6):73. doi: 10.3390/bioengineering8060073 (PMC8228610; doi:10.3390/bioengineering8060073)
Supplement: Supplementary file 1 [file bioengineering-08-00073-s001.zip › bioengineering-1231381-supplementary.pdf]

Supplementary material

Thermo-viscoelastic response of protein-based hydrogels

Aleksey D. Drozdov* and Jesper deClaville Christiansen

Department of Materials and Production

Aalborg University

Fibigerstraede 16, Aalborg 9220, Denmark

*E–mail: aleksey@m-tech.aau.dk

S-1 Chemical structure and preparation of hydrogels

S-1.1 Figure 1

Hyaluronic acid (HA) gels physically cross-linked by benzoxaborin-saccharide complexation were prepared by means of the following procedure (Figueiredo et al. [1]). First, sodium hyaluronate chains (molar mass 360 kg/mol) were end-functionalized separately with benzoxaborin derivative 2,1-BORIN (3,4-dihydro-2H-benzo[e][2,1]-oxaborinin-2-ol) and fructose (1-amino-1-deoxy-D-fructose) with the help of the amide coupling reactions. The gels were prepared by mixing (16 h at 4 °C) solutions of HA-2,1-BORIN and HA-fructose chains (mass fraction of polymer 15 g/L, 2,1-BORIN/fructose molar ratio 1:1) in 0.01M HEPES buffer with 0.15 M NaCl and various pH ranging from 6 to 9.

Shear oscillatory tests were conducted at room temperature with amplitude of oscillations $\epsilon_0 = 0.05$ and angular frequency ω ranging from 0.01 to 10 Hz.

S-1.2 Figures 2 and 3

Tetra-arm poly(ethylene glycol) (PEG) gels physically cross-linked by benzaldehyde-thiol complexation were prepared by using the following procedure (FitzSimon et al., [2]). First, propargyl cyanoacetamide (CA) was synthesized by reaction of propargyl amine with methyl cyanoacetate. PEG-CA chains were prepared by end-functionalization of 4-arm PEG-azide chains (molar mass 20 kg/mol) with cyanoacetamide via copper-catalyzed click reaction. Afterwards, two macromers (PEG-BCA and PEG-CBCA) were synthesized by attaching benzaldehyde and 4-cyanobenzaldehyde to PEG-CA chains by means of the Knoevenagel condensation reaction. To prepare hydrogels, PEG-BCA or PEG-CBCA macromers were mixed (total concentration of macromers 0.1) with a stoichiometric amount of 4-arm PEG-thiol chains (molar mass 10 kg/mol) in PBS buffer with pH = 7.4 at room temperature.

Rheological tests were conducted at room temperature. Shear oscillatory tests were performed with amplitude of oscillations $\epsilon_0 = 0.01$ and angular frequency ω ranging from 0.01 to 10 rad/s. Relaxation tests were performed with strain $\epsilon_0 = 0.01$. To study self-healing, a shear oscillation test was performed on a virgin disk sample. Then, the sample was cut into two equal pieces. The pieces were connected together, and, after a few minutes, the same test

was repeated.

S-1.3 Figures 4 and 5

Hydrazone cross-linked hyaluronic acid (HA) gels were prepared by means of the following procedure (Lou et al. [3]). First, sodium hyaluronate chains (molar mass 60 kg/mol) were end-functionalized by reaction with propargyl amine to produce hyaluronic acid-alkyne (HA-alkyne) chains. Afterwards, HA-alkyne chains were modified with hydrazine and aldehyde by reaction of HA-alkyne with azido-aldehyde or azido-hydrazine (12 % of carboxylate groups were replaced with aldehyde or hydrazine). HA-hydrazone gels were prepared by mixing dilute solutions of hydrazine-modified HA chains and aldehyde-modified HA chains in PBS with pH = 7.4 at 37 °C (at hydrazine to aldehyde ratio of 1:1) in the presence of 2-(aminomethyl)benzimidazole as a catalyst. Concentration of polymer in the pre-gel solution ϕ varied from 0.01 to 0.04, and molar fraction of catalyst $\phi_{\rm cat}$ varied from 0 to 100 mM.

Rheological tests were conducted in PBS buffer at temperature $T = 37$ °C. Shear oscillatory tests were performed with amplitude of oscillations $\epsilon_0 = 0.01$ and angular frequency ω ranging from 0.01 to 10 rad/s. Relaxation tests were performed with strain $\epsilon_0 = 0.1$.

S-1.4 Figure 6

PEG-DDI supramolecular gel cross-linked by hydrophobic interactions between fatty acids was prepared by means of the following procedure (Mihajlovic et al. [4]). First, segmented copolymer was synthesized by reaction between linear PEG chains (molar mass 8 kg/mol) and dimer fatty acid-based diisocyanate (DDI) (1 h at $T = 180$ °C followed by 30 min at $T = 200$ °C). After termination of the reaction with chloroform, the precipitated polymer was dried. Samples were compression-molded at temperature $T = 95$ °C under pressure of 100 bar. The gel (mass fraction of polymer $\phi = 0.25$) was prepared by immersing the compression-molded samples in an excess of water (24 h at room temperature).

Rheological tests were performed at temperature $T = 25$ °C. Shear oscillatory tests were conducted with amplitude of oscillations $\epsilon_0 = 0.001$ in the frequency range from 0.1 to 100 rad/s. Relaxation tests were performed with strain $\epsilon_0 = 0.001$.

S-1.5 Figure 7

Peptide-functionalized PEG gel was prepared by using the following method (Tunn et al. [5]). First, two peptides (acidic A_{4H3} and basic B_{4H3}) were synthesized (their amino acid sequences were provided in [5]) able to form coiled coil cross-links. These peptides were modified with cysteine residues to facilitate coupling to PEG chains. The modified peptides (concentration 10 mg/mL) were dissolved in PBS buffers at pH = 7.4 and coupled to 4-arm PEG-maleimide chains (molar mass 40 kg/mol) to form PEG-A_{4H3} and PEG-B_{4H3} macromers separately. Purified PEG-peptide conjugates were lyophilized and dissolved (concentration of 0.5 mM) in PIPPS buffers with pH = 8.1 at room temperature. The gel was prepared by mixing the solutions of PEG-A_{4H3} and PEG-B_{4H3} macromers in 1:1 proportion.

Rheological tests were conducted at temperature $T = 25$ °C. Shear oscillatory tests were performed with amplitude of oscillations $\epsilon_0 = 0.01$ in the range of frequencies between 0.01 and 100 rad/s. Relaxation tests were performed with strain $\epsilon_0 = 0.1$.

S-1.6 Figures 8 and 9

Three PEG-protein gels on the basis of EPE, L37I and L37V proteins cross-linked by coiled coils were prepared by using the following approach (Dooling and Tirrell [6]).

EPE proteins with a triblock architecture contain endblocks E (elastin-like polypeptides), a helical midblock domain P (derived from the cartilage oligomeric matrix protein), and N- and C-terminal cysteine residues. EPE, L37I and L37V proteins differ from one another by a single amino acid residue located at position 37 in the midblock P. Amino acid sequences of these proteins were given in [6]. The proteins were expressed in *E. coli* strain BL21 and purified by inverse temperature cycling.

The proteins were incorporated into networks by cross-linking the terminal cysteine residues with 4-arm poly(ethylene glycol) vinyl sulfone (PEG-VS). Lyophilized proteins were suspended in a phosphate buffer (concentration 150 mg/mL) with pH = 7.2. Tetra-arm PEG vinyl sulfone macromers (molar mass 10 kg/mol) were dissolved in triethanolamine (concentration 150 mg/mL). The solutions were mixed at a nominal 1:1 ratio of thiol to vinyl sulfone. Gelation was performed at room temperature (12 h) in the dark. The gels were swollen (48 h) in PBS

buffer with pH = 7.4 containing 0.02% of sodium azide. Mass fraction of proteins in the gels equaled 0.15.

Rheological tests were conducted at temperature $T = 25$ °C. Shear oscillatory tests were performed with the amplitude of oscillations $\epsilon_0 = 0.01$ in the interval of frequencies ω between 0.001 and 100 rad/s. Shear relaxation experiments (duration 2 h) were performed with strain $\epsilon_0 = 0.01$.

S-1.7 Figure 10

Protein gels $PC_{10}P$ and $PC_{30}P$ cross-linked by coiled coils were prepared by using the following technique (Olsen et al. [7]).

Proteins $PC_{10}P$ and $PC_{30}P$ were expressed by incorporation of the corresponding genes into *E. coli* strain SG13009. Telechelic protein sequences $PC_{10}P$ and $PC_{30}P$ (molar masses 20.5 and 36.6 kg/mol) were constructed from two helical end blocks (P) linked by 10 or 30 repeats of the nonapeptide sequence (C) served as flexible polyanionic linkers. Amino acid sequences of $PC_{10}P$ and $PC_{30}P$ proteins were provided in [7].

$PC_{10}P$ and $PC_{30}P$ gels (with concentration 0.07) were prepared by immersing lyophilized proteins into PBS buffer with pH = 7.6. The pre-gel solutions were held at 4 °C for 2 to 4 h until the proteins were fully hydrated. Then the solutions were heated above the sol-gel transition for 60 s and allowed to re-associate at room temperature.

Shear oscillatory tests were conducted at room temperature with the strain $\epsilon_0 = 0.1$ and frequencies ranged from 10^{-3} to 100 rad/s.

S-1.8 Figures 11 and 14

Protein gels P_4 were prepared by means of the following procedure ((Glassman et al. [8], Tang et al. [9]).

Coiled-coil protein P_4 has a multiblock architecture with the structure $C_{10}(P\ C_{10})_4$. It consists of four coiled-coil self-associating domains P on the protein backbone joined by flexible polyelectrolyte linkers C_{10}. Amino acid sequences of the blocks P and C_{10} were provided in [9]. The protein was expressed in *E. coli* and purified by ammonium sulfate precipitation and

anion exchange chromatography.

Protein gels with various mass fractions of proteins ϕ were prepared by hydration (2 days at 4 °C) of lyophilized P_4 proteins in sodium phosphate buffer with pH = 7.6. The specimens were annealed (2 h at 37 °C) in the dark and equilibrated (1 h) at the experimental temperature before testing.

Shear oscillatory tests were performed at temperature $T = 25$ °C (Fig. 11) and temperatures $T = 5$, 20, 35, 50 °C (Fig. 14) with strain amplitude $\epsilon_0 = 0.01$ in the interval of frequencies ω from 0.001 to 100 rad/s.

S-1.9 Figure 12

Protein CCE-CCK gels cross-linked by hetero-coiled-coil complexes were prepared by means of the following procedure (Sun et al. [10]).

The gels consist of two proteins CCE-$(GB_1)_4$-CCE and CCK-$(GB_1)_5$-CCK-$(GB_1)_5$-CCK (designated as CCE and CCK) phycally cross-linked by complexes formed by CCK and CCE blocks. Amido acid sequences of the globular protein GB_1 and the CCE and CCK polypeptides were reported in [10].

Protein gels with various mass fractions of proteins ϕ ranging from 40 to 160 g/L were prepared by mixing solutions of CCE and CCK proteins with equal molar fractions of CKK and CCE blocks in PBS with pH = 7.4 at room temperature.

Shear oscillatory tests were performed at temperature $T = 25$ °C with strain amplitude $\epsilon_0 = 0.01$ in the interval of frequencies ω from 0.1 to 100 rad/s.

S-1.10 Figures 16 and 17

Protein gels with o-Cys-P_4-Cys proteins were manufactured by using the following procedure (Tang et al. [11]).

Protein Cys-P_4-Cys is a modification of P_4 protein achieved by coupling the cysteine residues near the N- and C-termini. The protein was expressed in SG13009 strain of *E. coli* and purified by ammonium sulfate precipitation and anion exchange chromatography. Protein o-Cys-P_4-Cys was prepared by oxidation reaction (1 week at 4 °C) of Cys-P_4-Cys (mass fraction 0.1) in a

buffer with 6 M urea, 20 mM Tris and pH = 8.0. The oxidized proteins were dialyzed and lyophilized.

Protein o-Cys-P_4-Cys gels (with mass fractions 0.1 and 0.2) were prepared by hydration (2 days) of lyophilized proteins in PBS buffer followed by thermal treatment (heating to 90 °C and cooling down to 25 °C with the rate 5 K/min) to unfold the coiled-coil domains upon heating.

Shear oscillatory tests were performed with the strain amplitude $\epsilon_0 = 0.01$ in the interval of frequencies ω from 0.001 to 100 rad/s at temperatures $T = 15$, 25, 35 and 45 °C.

S-2 Supplementary Tables

Table S-1: Material parameters for HA gel cross-linked by benzoxaborin-saccharide complexation at various pH (Fig. 1).

pH	μ Pa	γ s^{-1}	Σ	K
6.0	9.80	63.0	0.10	0.0
7.4	21.60	1.21	1.06	0.28
9.0	154.85	0.23	1.93	0.42

Table S-2: Material parameters for PEG gels cross-linked by benzaldehyde-thiol complexation (Fig. 2).

Macromer	μ kPa	γ s^{-1}	Σ	K
PEG-BCA	2.39	0.13	0.42	0.27
PEG-CBCA	7.84	0.24	0.57	0.06

Table S-3: Material parameters for virgin and healed PEG gels cross-linked by benzaldehyde-thiol complexation (Fig. 3).

Gel	μ kPa	γ s^{-1}	Σ	K
Virgin	2.39	0.13	0.42	0.27
Healed	1.84	0.14	0.43	0.34

Table S-4: Parameters μ_1 and m in Eqs. (20) and (21).

Gel	μ_1 MPa	m	Figure
HA	0.54	1.65	5D
P_4	0.30	1.89	13A
CCK-CCE	0.05	1.44	13C

Table S-5: Material parameters for PEG-DDI gel (Fig. 6).

μ kPa	γ s^{-1}	Σ	K
27.75	0.18	3.33	0.98

Table S-6: Material parameters for peptide-functionalized PEG gel (Fig. 7).

μ kPa	γ s^{-1}	Σ	K
0.557	0.48	1.18	0.30

Table S-7: Material parameters for protein-functionalized PEG gels (Figs. 8 and 9).

Gel	κ	μ kPa	γ s^{-1}	Σ	K
PEG-EPE	0.684	14.12	0.11	2.89	0.130
PEG-L37I	0.640	12.01	1.12	2.42	0.078
PEG-L37V	0.514	10.63	6.10	1.88	0.019

Table S-8: Material parameters for telechelic protein gels (Fig. 10).

Gel	μ kPa	γ s^{-1}	Σ	K
$PC_{10}P$	4.22	0.0087	0.71	0.72
$PC_{30}P$	1.28	0.0010	2.56	182.0

Table S-9: Activation energies $E_{a\gamma}$ and E_{aK} for P_4 protein gel (Fig. 15).

$E_{a\gamma}$ kJ/mol	E_{aK} kJ/mol
74.8	62.7

Table S-10: Activation energies $E_{a\gamma}$ and E_{aK} for o-Cys-P_4-Cys protein gels (Fig. 18).

ϕ	$E_{a\gamma}$ kJ/mol	E_{aK} kJ/mol
0.1	62.0	57.6
0.2	94.5	84.1

References

[1] Figueiredo, T.; Jing, J.; Jeacomine, I.; Olsson, J.; Gerfaud, T.; Boiteau, J.-G.; Rome, C.; Harris, C.; Auzely-Velty, R. Injectable self-healing hydrogels based on boronate ester formation between hyaluronic acid partners modified with benzoxaborin derivatives and saccharides. *Biomacromolecules* **2020**, *21*, 230–239.

[2] FitzSimons, T.M.; Oentoro, F.; Shanbhag, T.V.; Anslyn, E.V.; Rosales, A.M. Preferential control of forward reaction kinetics in hydrogels crosslinked with reversible conjugate additions. *Macromolecules* **2020**, *53*, 3738–3746.

[3] Lou, J.; Liu, F.; Lindsay, C.D.; Chaudhuri, O.; Heilshorn, S.C.; Xia, Y. Dynamic hyaluronan hydrogels with temporally modulated high injectability and stability using a biocompatible catalyst. *Adv. Mater.* **2018**, *30*, 1705215.

[4] Mihajlovic, M.; Staropoli, M.; Appavou, M.-S.; Wyss, H.M.; Pyckhout-Hintzen, W.; Sijbesma, R.P. Tough supramolecular hydrogel based on strong hydrophobic interactions in a multiblock segmented copolymer. *Macromolecules* **2017**, *50*, 3333–3346.

[5] Tunn, I.; Harrington, M.J.; Blank, K.G. Bioinspired histidine–Zn^{2+} coordination for tuning the mechanical properties of self-healing coiled coil cross-linked hydrogels. *Biomimetics* **2019**, *4*, 25.

[6] Dooling, L.J.; Tirrell, D.A. Engineering the dynamic properties of protein networks through sequence variation. *ACS Cent. Sci.* **2016**, *2*, 812–819.

[7] Olsen, B.D.; Kornfield, J.A.; Tirrell, D.A. Yielding behavior in injectable hydrogels from telechelic proteins. *Macromolecules* **2010**, *43*, 9094–9099.

[8] Glassman, M.J.; Chan, J.; Olsen, B.D. Reinforcement of shear thinning protein hydrogels by responsive block copolymer self-assembly. *Adv. Funct. Mater.* **2013**, *23*, 1182–1193.

[9] Tang, S.; Wang, M.; Olsen, B.D. Anomalous self-diffusion and sticky Rouse dynamics in associative protein hydrogels. *J. Am. Chem. Soc.* **2015**, *137*, 3946–3957.

[10] Sun, W.; Duan, T.; Cao, Y.; Li, H. An injectable self-healing protein hydrogel with multiple dissipation modes and tunable dynamic response. *Biomacromolecules* **2019**, *20*, 4199–4207.

[11] Tang, S.; Glassman, M.J.; Li, S.; Socrate, S.; Olsen, B.D. Oxidatively responsive chain extension to entangle engineered protein hydrogels. *Macromolecules* **2014**, *47*, 791–799.
